# A Survey of Current Practice in Operative Management of Testicular Torsion in Poland

**DOI:** 10.3390/children10040643

**Published:** 2023-03-30

**Authors:** Aneta Piotrowska-Gall, Piotr Stępień, Przemysław Wolak

**Affiliations:** 1 Collegium Medicum, Jan Kochanowski University, 25-369 Kielce, Poland; 2 Department of Pediatric Surgery, Urology and Traumatology, Provincial Hospital in Kielce, 25-736 Kielce, Poland

**Keywords:** testicular torsion, acute scrotum, testis, orchiectomy, management

## Abstract

The primary aim of this study is to evaluate heterogeneity in the current management of testicular torsion (TT). A secondary aim is to investigate incidences of recurrent torsion and the methods used for primary fixation. An online multiple-choice questionnaire comprising 10 questions was distributed to paediatric surgeons and urologists. There were a total of 99 questionnaires distributed to representatives from 39 paediatric surgery and urology departments in Poland. The majority of participants agreed on fixation of the torsed testicle (98%). Use of sutures was reported by 95% of surgeons: absorbable by 48%, non-absorbable by 42%, and 4% using both. There was no consensus on the number of sutures. The contralateral testicle was always fixed by 69%, with 28% fixing it only in case of necrosis and amputation of the torsed testicle, and the remaining 2% never fixing the contralateral side. In case of negative scrotal exploration, 18% of surgeons would still fix the testis. The recurrence of torsion after prior fixation was identified by eight of the participants. The most commonly reported primarily used technique was absorbable sutures. There is a general consensus on the fixation of torsed testicles; however, other aspects remain controversial. Based on the survey and the literature review, the use of non-absorbable sutures rather than absorbable sutures would be advisable.

## 1. Introduction

Testicular torsion (TT) nowadays is known as an emergency condition requiring prompt diagnosis and surgical intervention. However, it was only in 1895 when Hutchinson suggested that this condition should be referred to a surgeon rather than a psychiatrist [1]. Over the years, the approach to this condition has changed and new concepts have emerged. Its prevalence varies depending on the age of the patient. Reports show the incidence of 2.9–4.5 cases per 100,000 people each year [2,3,4]. The aetiology of TT remains unknown. There are suspected risk factors, such as the ‘bell clapper’ deformity, changes in the weather, and family history or gene mutations [1,4].

It is widely accepted that suspected TT requires urgent surgical intervention. The severity of consequences resulting from torsion depends on two key factors: the degree of torsion, which can range from 180 to 1080 degrees, and the time taken to untwist the testicle. According to Zvizdic’s research on the duration of symptoms, the latter factor is particularly crucial in determining the extent of damage. Time is a critical aspect, as irreversible changes may occur within just 4 h of symptom onset [5]. Delays in providing prompt and appropriate treatment can result in necrosis of the testicle and impaired fertility, and bilateral torsion is a particularly feared complication as it can lead to infertility and hormonal disturbances. The choice of operative procedure is the responsibility of the leading surgeon, which includes scrotal exploration, untwisting the torsed spermatic cord, and orchiectomy or orchidopexy, depending on the viability of the organ. Many aspects of the management of suspected TT are still debated. There are different views and opinions regarding the methods of fixing the testicle, as well as the necessity for contralateral exploration and fixation.

The primary aim of this study is to evaluate the heterogeneity in the current management of TT in Poland. A secondary aim is to investigate incidences of recurrent torsion and the methods used for primary fixation.

## 2. Materials and Methods

A recruitment email was sent to paediatric surgeons and paediatric urologists practising in Poland, with a link to an online survey. The questionnaire was distributed to all departments of Paediatric Surgery in Poland via the National Consultant of Paediatric Surgery, the Polish Paediatric Surgeons’ Association, and e-mail requests. The survey was taken online, open (non-anonymous), and voluntary. All participants were informed of the purpose of the study. The respondents were asked to answer questions according to their practice. The survey included 10 questions concerning methods of operation, fixation preferences, management of the contralateral testis, removal of an appendix of testis, and recurrence rates of scrotal explorations. Questions were multiple choice and some of them contained an additional comment section to allow participants to add supplementary information. Only respondents that completed the survey were included in the study. The questionnaire is included in Appendix A.

## 3. Results

A total of 99 questionnaires were issued. Participants represented 39 out of 60 departments of paediatric surgery in Poland. The complete respondent demographic is displayed in Table 1. A total of 35 respondents were paediatric surgery residents, and 64 were paediatric surgery specialists, including 15 heads of departments. Within the group, there were nine surgeons with paediatric urology specialisation. The majority (61%) of respondents were male and were practising in academic institutions (58%). Table 2 summarises the surgical approach to a patient with TT.

### 3.1. Management of Torsed Testicle

The majority of respondents (98%) fix the testicle after torsion. However, only three (3%) participants rely entirely on sutureless fixation of the testis. As a result, 94 (95%) respondents used sutures to fixate the torsed testicle. However, there was no consensus on the type of sutures used. Absorbable sutures were used by 48 (48%) surgeons, while non-absorbable sutures were used by 42 (42%), and 4 (4%) surgeons used both types. Similarly, the respondents also disagree on the number of sutures used, with the most common response being two sutures for fixation (57%).

### 3.2. Management of Contralateral Testicle

A total of 69 participants always fixate the contralateral testicle, with the remaining 28 fixating it only in the case of necrosis and amputation of the torsed testicle, and 2 respondents never fixate the contralateral testis. There is also a discrepancy in timing of the fixation. The majority prefer to perform a one-step procedure, while the others opt for scheduling an elective surgery within a few weeks of the main event. As a result, according to our survey, 30% of surgeons perform contralateral orchidopexy as a subsequent procedure. There are three approaches described by participants to gain access to the torsed testicle: via individual incisions on both sides (80%), through the septum (17%), or through a midline incision (1%).

### 3.3. Additional Manoeuvres

Only four (4%) respondents would not remove the Morgagni appendix during exploration, while the remaining surgeons routinely excise it. 

If the diagnosis during the operation reveals a condition other than torsion, 18% of the surveyed surgeons would still opt for testicular fixation. Additionally, some participants (5%) stressed the importance of fixation when an anatomical anomaly is detected. Three surgeons reported an inconsistent approach to managing normal testicles without torsion during operation.

### 3.4. Re-Operations 

The need for re-exploration of the testicle after previous surgery for TT was reported by fifteen surgeons. Recurrences of torsion after prior fixation were identified by twelve of the participants. The techniques used in previous cases were as follows: absorbable sutures were used in six cases, including two participants from the same centre; non-absorbable sutures were used in four cases, including two participants from the same centre; sutureless fixation was used in one case; and there was no data available on the fixation method used in one case. Two surgeons reported the need for re-operation due to bleeding or haematoma, while another two had observed abscess formation. In three cases there was testicle atresia observed and amputation was carried out.

## 4. Discussion

In light of the fact that there are no national recommendations to follow, the care of patients with suspected TT is highly dependable on the decision-making surgeon. Although the EAU’s ESPU Guidelines exist, they are not universally followed, and the management of TT can vary between healthcare facilities. While the development of TT and the best treatment algorithms are still debated, new data continue to surface in the literature.

It is well known that prolonged ischemia time will cause irreversible injury and atrophy of the gonad involved [6]. In a recent animal study by Xia et al., it was shown that unilateral testicular torsion causes impairment not only to the affected testicle, but also decreases the function of the contralateral one due to the ischemia-reperfusion mechanism and later autoimmune response [7]. In the case of suspected TT, immediate exploration and restoration of blood flow are indicated. Some studies showed improved salvaging of the organ after manual detorsion [8]. 

### 4.1. Management of Torsed Testicle

It is generally agreed that emergency surgical exploration is mandatory if a diagnosis of TT cannot be ruled out. To prevent re-torsion, fixation is advised. This statement is in agreement with our survey results (98%). However, there is no consensus on how the fixation should be achieved. In the literature, a variety of descriptions of methods used to fixate the torsed testicle are present. In our survey, most of the participants (95%) use suture fixation. The efficacy of fixation techniques was assessed in several animal studies [9,10,11]. Morse et al. presented on the animal model that secure fixation may be accomplished by the creation of a broad area of dense adhesions. The researcher proposed a four-point fixation technique with a window in the tunica vaginalis based on the finding of the study. Additionally, the comparison of silk and chromic gut sutures showed that the silk sutures formed more adhesions and there was no abscess observed, unlike in cases with chromic gut sutures [9]. Similar results were achieved in a study by Bellinger et al., where it was confirmed that simple suture fixation results in the creation of adhesions, with nylon sutures causing less severe changes than in chromic-fixed testes. Active spermatogenesis saw a decrease in 47% of subjects due to severe acute inflammation or abscess formation. In the group of dartos-fixed testes, complete circumferential adherence was observed, along with only minor histological changes in the affected testis [10]. Our survey shows that 48% of participants use absorbable sutures and 42% use non-absorbable sutures, while only 8% of respondents use sutureless methods alone or in combination with sutures. Mor et al. studied eight patients with recurrent torsion involving the ipsilateral or the contralateral gonad, presented 6 months to 23 years after initial testicular fixation due to the testicular torsion. They analysed surgical technique and suturing materials where, in seven cases, absorbable sutures were used. They emphasised that the lack of consensus concerning the best fixation method may put a considerable population at risk of recurrent torsion [12]. According to the findings of a comprehensive study that was carried out by Moore and colleagues, there is no single method that is superior to the others for doing orchidopexy in TT. The incidence of surgical complications, such as ipsilateral atrophy rate and abscess formation, was found to have ranged from 9.1% to 47.5% in their research [13].

Another controversial aspect is the number of sutures used to fix the testicle. According to the results of our survey, the most frequent response was two-point fixation, which was chosen by 57% of the participants. To our knowledge, there are no data showing a comparison of the advantages and risks of using a certain number of sutures. However, the existing reference literature recommends three-point fixation using non-absorbable sutures [11,14,15,16,17]. Data have shown concerns regarding the damaging effects on the blood-testis barrier. When the barrier is violated, higher levels of anti-sperm antibodies are detected, leading to spermatogenesis disorders. Therefore, the literature advises the use of sutureless fixation as a method to avoid violating the blood-testis barrier and its potential effects on fertility, as well as the increased risk for testicular malignancy [17]. 

### 4.2. Management of Contralateral Testicle

The majority of participants in the study (70%), shared a view of the need for routine contralateral exploration and fixation of the testis. The necessity of the contralateral fixation is usually explained by a high incidence of bilateral bell clapper deformity and therefore, the risk of anorchia [5,16]. The EAU Guidelines on Paediatric Urology also recommend the fixation of contralateral testis [5].

There are also reports of bilateral synchronous torsion identified in newborns with minimal clinical manifestation [18]. However, in a survey by Abdelhalim et al., the need for contralateral testis fixation in neonatal torsion is questioned. In the event of prenatal torsion, contralateral fixation was performed by 84.9% of respondents. Additionally, there was no consensus on contralateral fixation in undescended testis (in prepubertal boys, 79.2%, and postpubertal boys, 81.13%) [19]. These findings contradict the results reported in the literature, which suggest a tenfold increased risk of torsion in individuals with cryptorchidism [1]. 

However, this management was called into question by Arnbjornsson et al. It was presented in the study that during the observation period (mean: 7 years), no contralateral torsion was reported. Some have argued that in cases where the risk of contralateral torsion is low, the potential harm caused by contralateral fixation outweighs its benefits [20]. 

Djahangirian et al. reinforces that testis fixation may lead to complications, as he reported in the study that 18% of patients had postoperative complications, such as recurrent hydrocele, wound infection, or urinary tract infection. In addition, six patients developed ipsilateral testicular atrophy [21].

One of the predisposing factors for TT described in the literature is a bell clapper deformity [1]. In this condition, parietal lamina of the tunica vaginalis is not attached to either testis or epididymis, where, as a result, the testicle hangs freely. In the study conducted by Martin et al., the prevalence of the bell clapper anomaly in a group of patients with nonpalpable testis and a group of patients with testicular torsion were compared. In 50 cases with ipsilateral nubbins, a contralateral fixation was performed, revealing one case of partial anomaly. In the second group, in 21 of 27 cases, the bell clapper deformity was also observed in the contralateral testicle [16].

Caesar et al. presented in his autopsy series that the incidence of the bell clapper deformity was 12%, suggesting that other factors must be involved in addition to the anatomic anomaly [22].

Notably, a higher percentage (68%) of our respondents favoured contralateral exploration upon initial exploration. Some respondents delayed the timing of contralateral fixation surgery, arguing that prolonged torsion can cause excessive inflammatory reactions in the scrotum, and therefore the surgery should be performed at a later stage. However, this approach requires an additional hospital stay and procedure under general anaesthesia, which carry relative risks. Furthermore, a recent study by Hampl et al. [23] reported that bacterial growth in the tunica vaginalis cavity of patients who underwent orchiectomy due to testicular torsion was not observed in the vast majority of cases, and wounds completely healed within 90 days after surgery.

The recent study by Koh et al. reported a low incidence of postoperative complications in exploration of the other side due to testicular appendage torsion. The study also noted that selecting a midline raphe incision may allow for simultaneous bilateral exploration [24]. However, some surgeons choose to use individual incisions on both sides of the scrotum, as showed in our survey, arguing that it is performed to avoid infecting the healthy part and to achieve better visualisation during the surgical procedure.

### 4.3. Additional Manoeuvres

A vast majority (96%) of surgeons in our survey routinely excise the appendix testis in order to pre-empt torsion of the appendix in the future. Similar results were achieved in a survey on operative management of TT in the UK and Ireland, by Bolln et al. [25]. 

There are no guidelines regarding the management of the testis in negative exploration, where the testis is found to be non-torsed. In our survey, 18% of respondents would proceed with fixation, while another 8% would consider it under special circumstances, such as the presence of an anatomical anomaly. In the literature, there is no consistency regarding management [25,26]. 

Naumann et al. describe orchidopexy during negative exploration as an unnecessary procedure, which is performed due to the lack of guidelines and proper training. In this study, 57% of patients without a diagnosis of TT underwent ipsilateral orchidopexy, and 14% underwent contralateral orchidopexy. Orchidopexy was more often performed by general surgeons than by urologists [27].

### 4.4. Re-Operations

There are reported complications after exploration and fixation of the torsed testis, including haematoma, abscess formation, and atrophy of the testis. In our survey, six surgeons reported re-operations due to complications. In some studies, it is shown that up to 54% of patients develop testicular atrophy after TT [21,28,29]. Proper assessment of the risk of complications may help guide decision making for the management of TT [30].

It is important to note that orchidopexy might not guarantee the prevention of potential future torsion, and thus might give doctors and patients a false feeling of safety. There are numerous reports of re-torsion in the literature. Those studies demonstrate a correlation between the recurrence of torsion and the use of absorbable sutures [12,25,29].

### 4.5. Other Considerations

Various studies on animals have suggested that unilateral TT may cause damage to the contralateral testicle [17,31]. Furthermore, some studies have suggested that testicular torsion may lead to decreased fertility. This hypothesis posits that ischemia/reperfusion injury, autoimmunisation, and alterations in biochemical and neurohormonal pathways contribute to this phenomenon [17,31,32,33]. 

The primary reasons for follow-up in patients after TT are potential fertility and hormonal disturbances, although the exact consequences remain inconsistent in published data, as noted by the EAU guidelines [5]. Almekaty et al. conducted a study on post-pubertal patients who underwent TT, which revealed that only 32% of the participants had normal sperm parameters. In contrast, 34% had azoospermia, and 34% showed abnormal seminal analysis. However, it is hypothesised that sperm may survive for a period of time in an ischemic testis. This is supported by the fact that in orchiectomy specimens, sperm retrieval was successful in 47% of cases [17]. Interestingly, in a recent study by Gielchinsky et al., the pregnancy rate in 63 couples, where males had experienced TT, was on a comparable level to the general population [34]. 

#### Limitations of the Study

Our study has some limitations which must be acknowledged. First, the ability to gain access to the wide scope of surgeons from all hospitals and regions practising in Poland was limited. Taking that into account, the people who responded to these survey questions may not be a truly random sample. Second, our population was restricted only to paediatric surgeons and paediatric urologists, and thus there is a potential for sampling bias. It is important to note that general surgeons or urologists, who were not included in this study, may have different approaches to treatment. The study is also limited to surgeons from Poland; therefore, it would be beneficial to compare the data with professionals in other countries.

Our data validity, as with any self-reported survey, may potentially be threatened by response biases, where the format of the question or the nature of the previous questions may have had an unwanted impact on how a person responded to the survey.

Despite these limitations, we believe that our findings are meaningful.

First, to our knowledge, there is no prior study exploring the heterogeneity in the clinical management of TT in Poland. Second, the variety of responses and collected literature data reveal that there is a need for further studies regarding the proper management of TT, in order to preserve reproductive capabilities.

## 5. Conclusions

In conclusion, this survey demonstrates a wide variation in practices of TT management amongst paediatric surgeons in Poland. The emergency procedure for TT is highly dependent on the decision of the caring surgeon. The most controversial aspect is the method used for the fixation and management of the contralateral testis. There is also a lack of consensus in the literature regarding evidence-based practice. There is an indispensable need for future studies on multi-centre and long-term outcomes of the effects on the testicles after an incident of torsion. To provide the best patient care, all of the benefits and risks must be weighed with proper counselling of patients and their parents in order to facilitate a better understanding. Therefore, there is a need to establish national guidelines in order to provide homogenous management of TT.

## Figures and Tables

**Table 1:** Table presenting respondent demographic.

N = 99	
Paediatric Surgery Resident	35 (35%)
Paediatric Surgery Specialist	64 (65%)
Head of Department	15 (15%)
Paediatric Urology Specialist	9 (9%)
Male	60 (61%)
Female	39 (39%)
Practice environment	
Academic Institution	58 (59%)
Regional Hospital	20 (20%)
Other	21 (21%)

**Table 2:** Table presenting surgical approach to the patient with TT.

Fixation of torsed testicle	97 (98%)
Fixation with suture	94 (95%)
Absorbable	48 (48%)
Non-absorbable	42 (42%)
Usage of both types	4 (4%)
Sutureless fixation	
Combined with sutures	5 (5%)
Only sutureless	3 (3%)
Without fixation	2 (2%)
Number of sutures	
2	56 (57%)
3	31 (31%)
4	3 (3%)
Variable (2–5)	4 (4%)
Fixation of contralateral normal testis	
Always	69 (70%)
Only in case of TT with gangrene	28 (28%)
Never	2 (2%)
Timing of fixation of the contralateral normal testis	
At the initial exploration	67 (68%)
Elective at later surgical session	30 (30%)
Never	2 (2%)
Contralateral access	
Individual incision on both sides	79 (80%)
Through midline raphe	17 (17%)
Midline incision	1 (1%)
Amputation of Morgagni appendix	
Yes	95 (96%)
No	4 (4%)
Fixation without torsion	
Yes	18 (18%)
No	77 (78%)
Sometimes	3 (3%)
When anatomical anomaly present	5 (5%)
Reported re-operation	
Yes	15 (15%)
No	77 (77%)
Maybe	8 (8%)
Estimated number of observed cases per year	
<10	27 (27%)
10–30	44 (44%)
30–50	3 (3%)
No data	24 (24%)

## Data Availability

The data presented in this study are available on request from the corresponding author. The data are not publicly available due to privacy restrictions.

## Appendix A

Questionnaire (translated into English)

Name:

Hospital:

1. Fixation of the torsed testicle:
  - Suture fixation, absorbable
  - Suture fixation, non-absorbable
  - Sutureless fixation, Jaboulay procedure
  - Sutureless fixation, Dartos pouch
  - Other, please specify
2. In case of suture fixation, number of sutures used:
  - 2
  - 3
  - 4
  - Other, please specify
3. Contralateral fixation:
  - routinely, single procedure
  - routinely as elective procedure
  - only in case of necrosis and orchidectomy, single procedure
  - only in case of necrosis and orchidectomy, elective procedure
  - other, please specify
4. Contralateral access:
  - through midline raphe
  - Individual incision on both sides
  - Other, please specify
5. Amputation of Morgagni appendix
  - routinely during the procedure
  - no
  - other, please specify
6. Fixation of the testis if the diagnosis at exploration is not torsion:
  - yes
  - no
  - other, please specify
7. Have you ever had a patient requiring re-operation after testicular torsion:
  - yes
  - no
  - I am not sure
  - other, please specify
8. If yes, what was the method of primal fixation;
  - Suture fixation, absorbable
  - Suture fixation, non-absorbable
  - Sutureless fixation, Jaboulay procedure
  - Sutureless fixation, Dartos pouch
  - Other, please specify
9. Please specify the cause and the outcomes of the reoperation: -
10. Approximate number of cases per year:
  - <10
  - 10–30
  - 30–50
  - >50
  - I am not able to determine
  - other, please specify
